# Examining the Effect of Online Engagement on Older Adults’ Subjective Memory Capability: Cross-Sectional Path Analysis

**DOI:** 10.2196/73018

**Published:** 2026-05-06

**Authors:** Soohyoung Rain Lee, Hang Liu

**Affiliations:** 1 Wurzweiler School of Social Work, Yeshiva University, 2495 Amsterdam Ave, 905, New York, 10033, United States, 1 9173498419; 2 Selfhelp, New York, NY, United States

**Keywords:** online engagement, homebound older adults, subjective memory ability, social relationships, digital inclusion

## Abstract

**Background:**

The utility of online engagement in enhancing quality of life and mitigating social isolation among older adults is well documented. However, its relationship with cognitive functioning, particularly through online engagement, requires further exploration.

**Objective:**

This study investigated whether active online engagement via the Virtual Senior Center (VSC) program was associated with subjective memory capability among older adults and whether subjective memory capability was associated with psychological well-being and loneliness.

**Methods:**

This study included a cross-sectional sample of 53 homebound older adults participating in the VSC program, which offers diverse online classes to promote social interaction. Path analysis was conducted to examine the associations among online engagement, subjective memory capability, quality of life, and loneliness.

**Results:**

Increased participation in VSC activities was associated with higher subjective memory capability (β=0.29, 95% CI 0.04-0.54; *P*<.02). Subjective memory capability was associated with better quality of life (β=0.29, 95% CI 0.04-0.54; *P*<.001) and lower loneliness (β=0.29, 95% CI 0.04-0.54; *P*<.003). No direct associations were observed between online engagement and quality of life or loneliness.

**Conclusions:**

Subjective memory capability was associated with better quality of life and lower loneliness. Although online engagement had no direct association with quality of life and loneliness, the observed indirect pattern suggests that subjective memory capability may represent a psychologically meaningful pathway through which structured online engagement relates to well-being. These findings highlight the potential of digital platforms to complement traditional forms of socialization, particularly for older adults facing physical or geographic barriers to interaction.

## Introduction

### Background

In an increasingly digital society, online engagement, which is defined as active participation in digital platforms for social interaction, information sharing, and learning, has emerged as a key factor influencing the well-being of older adults. Given the potential of digital health technologies to mitigate social isolation and promote cognitive health, understanding the impact of online engagement on cognitive function is critical, particularly for homebound or mobility-challenged older adults [1,2].

Existing research has reported that active online engagement is associated with reduced feelings of isolation, which are prevalent among older adults, and has been examined in relation to cognitive functions such as memory. For example, older adults who use online platforms for social interaction have reported higher perceived quality of life and cognitive function [3,4]. However, there remains a notable gap in understanding the association between online engagement and subjective memory capability, defined as an individual’s self-assessment of everyday memory functioning, and how these factors relate to overall well-being among older adults [5,6]. Many previous studies have focused on general internet use or access rather than participation in structured, program-based online engagement designed to promote sustained social and cognitive interaction [7,8]. As digital health and online community-based interventions continue to expand, understanding how structured engagement contexts relate to cognitive self-perception and well-being represents an important area for further investigation.

Given the growing interest in digital therapeutics and telehealth interventions, it is important to explore how online engagement contributes to cognitive self-perception and well-being among older adults. This study examined the association between participation in the Virtual Senior Center (VSC) program and subjective memory capability and social well-being among homebound older adults. Specifically, this research aimed to answer the following questions:

1. Does participation in a VSC influence older adults’ subjective memory capability?
2. Does increased online engagement lead to improved social connectedness and well-being?
3. How does self-perceived memory capability mediate the relationship between online engagement and well-being?

It was hypothesized that greater participation in VSC activities would be positively associated with subjective memory capability and that subjective memory capability would be associated with higher quality of life and lower loneliness. This study contributes to the broader discussion of digital inclusion and cognitive health interventions by examining these relationships within a structured online engagement program for a homebound population. By focusing on subjective memory capability as a psychological mechanism, this study offers practical insights for designing digital health interventions and community-based programs targeting socially isolated older adults.

### Literature Review

Research on internet communication technology in aging populations has highlighted the potential of online engagement to enhance social interaction, reduce isolation, and support psychological well-being [7,9]. Older adults who actively use online platforms for social interaction have reported higher life satisfaction, improved mental health, and better perceived cognitive outcomes than those who remain digitally disconnected [8,10].

Despite the recognized benefits of online engagement for mental health, studies examining its relationship with cognitive function and memory perception remain limited. Much of the literature has focused on the psychosocial aspects of general internet use, such as reducing loneliness and enhancing social connectedness [11,12]. Less attention has been paid to structured, program-based online engagement designed to promote sustained social and cognitive interaction. More recent studies suggest that such structured online engagement may be associated with cognitive benefits, including subjective memory capability and other indicators of cognitive self-perception [13,14].

The VSC program in this study was developed to provide older adults with structured online activities, including educational courses, group discussions, and interactive social experiences. Unlike passive internet browsing or unstructured social media use, the VSC emphasizes scheduled and socially interactive participation. As a community-based platform, it facilitates ongoing engagement among older adults who may face barriers to in-person interaction. Specifically, the facilitator leads each class and encourages participants to engage and lead the group. In contrast to studies focusing on general internet use, the VSC represents a structured program-based approach to online engagement, making it a useful context for examining how sustained participation in organized digital activities relates to cognitive self-perception and well-being.

Prior studies have reported that tailored digital interventions may support cognitive engagement among older adults. For example, the Internet-Based Conversational Engagement (I-CONECT) program found that structured online socialization significantly improved cognitive performance in older adults [13,14]. In a similar way, the VSC reflects an interactive, community-based approach that may be relevant to digital health interventions targeting older adults who face barriers to in-person engagement.

### Theoretical Framework

This study is grounded in 2 complementary theoretical frameworks that help explain how online engagement may relate to cognitive self-perception and well-being in older adults. Cognitive reserve theory posits that engaging in intellectually stimulating activities helps build cognitive resilience, thereby maintaining cognitive function and delaying the onset of cognitive decline [15]. Research has shown that older adults who participate in structured cognitive and social activities, such as online discussions, memory training, and digital learning platforms, demonstrate better cognitive performance and self-perceived memory capabilities [13,14]. Digital interventions, including internet-based cognitive training and virtual social engagement, have been found to foster cognitive reserve, particularly among older individuals at risk of social isolation [1,2].

**Figure 1.**
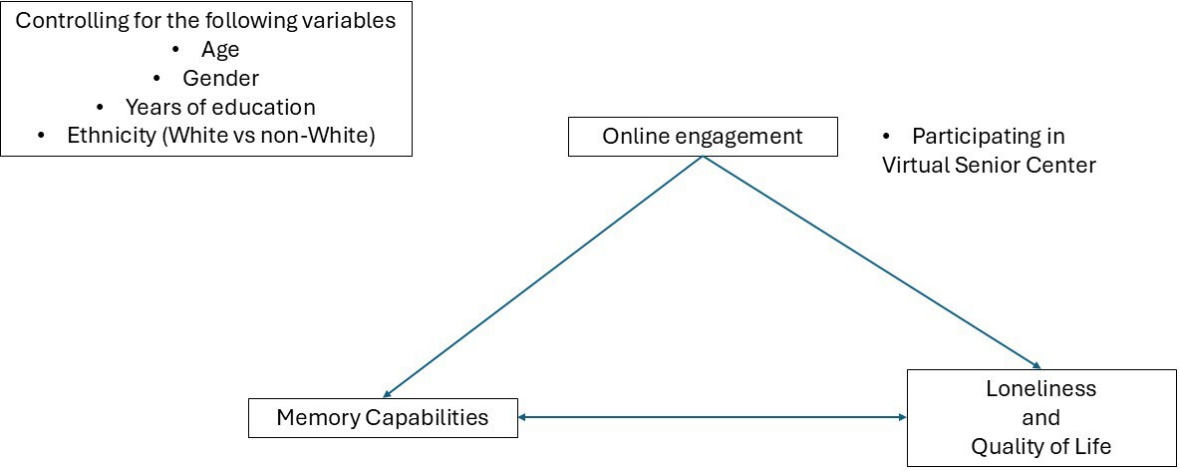
Theoretical framework.

Complementing this perspective, socioemotional selective theory suggests that, as individuals age, they become more selective in their social interactions, prioritizing emotionally meaningful relationships and experiences [16]. Studies have demonstrated that older adults tend to prefer digital communication tools that facilitate socially enriching interactions, such as video calls and virtual communities, over passive internet browsing or impersonal interaction [3,4]. Online platforms such as the VSC cater to these preferences by providing structured, interactive environments where participants can engage in meaningful social and cognitive activities. Research on digital health interventions has highlighted that programs designed around socioemotional needs and cognitive engagement, such as the I-CONECT program, are particularly effective in maintaining cognitive function and improving emotional well-being in older populations [13,14] (see Figure 1 for the theoretical model).

Given the increasing importance of digital inclusion, addressing disparities in digital access and engagement is critical [17]. Socioeconomic status, health conditions, age, and living arrangements all influence older adults’ ability to benefit from online engagement [9,18-20]. This study examines the role of online engagement in supporting older adults who choose to age in place, particularly those with physical impairments that limit their access to in-person socialization. By facilitating virtual social interactions and providing access to cognitively stimulating activities, online engagement has the potential to enhance subjective memory capability and overall quality of life for these individuals.

By integrating cognitive reserve theory and socioemotional selective theory, this study provides a comprehensive framework for understanding the dual role of digital engagement in promoting cognitive self-perception and emotional well-being among older adults.

## Methods

### Participants and Recruitment

This study examined a cohort of 53 community-dwelling, homebound older adults participating in the VSC program, an online engagement platform designed to support social interaction and cognitive health. Participants were recruited through community aging services, older adult centers, and digital literacy outreach programs targeting older adults at risk of social isolation due to physical impairments, socioeconomic constraints, or mobility limitations.

These partnering organizations identified and referred individuals who experienced difficulty participating in in-person social activities, including those with limited mobility or chronic health conditions. Therefore, they represent a convenience sample of individuals enrolled in the VSC program during the study period.

To be eligible for the study, participants had to (1) be aged 55 years or older and residing in a community setting, (2) have access to a computer with internet connectivity (provided if necessary), (3) be enrolled in the VSC program and actively participating in its activities, and (4) report no clinically diagnosed severe cognitive impairments or neurodegenerative conditions (eg, Alzheimer disease).

Although the program primarily serves older adults aged 55 years or older, the final sample included participants ranging from 47 to 98 years, reflecting real-world enrollment patterns within the VSC program (ie, individuals with disabilities). This study focused on older adults who were dual eligible for Medicaid and Medicare, a population often characterized by low income, greater medical complexity, and increased health vulnerability [21-24]. To ensure equitable participation, participants were provided with computers; internet access when needed; and initial onboarding support, including assistance with logging into sessions and basic troubleshooting. When participants experienced technical difficulties, staff members could resolve the issues remotely without physically visiting their location to expedite the resolution process.

### Study Design and Measurement

This study used a cross-sectional design, collecting data over a 6-month period (August 2017-January 2018) using a structured online questionnaire distributed through the VSC platform. Participants completed the survey after at least 3 months of engagement with the program to ensure that they had sufficient exposure to VSC activities before reporting on their experiences.

### Ethical Considerations

This study was approved by the WCG institutional review board affiliated with Yeshiva University (IRB00023382). All participants provided informed consent prior to participation. Participant privacy and confidentiality were strictly maintained throughout the study, and all data were de-identified prior to analysis.

### Measurement

#### Subjective Memory Capability

Participants’ self-perceived memory function was assessed using the Metamemory in Adulthood (MIA) capacity subscale [25-27]. The MIA measures self-assessed memory performance in areas such as name recall, event tracking, and appointment remembering (eg, “I am good at remembering names,” “I am good at remembering birth dates,” and “I have no trouble keeping track of my appointments”). This validated measure has been widely used in aging research and has demonstrated moderate correlations with objective cognitive function [28].

#### Loneliness and Social Engagement

Loneliness was measured using the University of California, Los Angeles (UCLA), Loneliness Scale–Short Form [29,30], which assesses perceived social connectedness and feelings of isolation (eg, “I feel lack of companionship” and “I feel left out”). Given that older adults in this study were homebound, the short version of the UCLA scale was selected to reduce respondent burden while maintaining validity [31].

#### Quality of Life

Quality of life was measured using the Older People’s Quality of Life Questionnaire (eg, “I enjoy my life,” “I am happy most of the time,” and “I feel lucky compared to most people”) [32-34]. This scale captures emotional well-being, social relationships, and life satisfaction, making it a comprehensive measure for assessing the impact of online engagement.

#### Online Engagement

Online engagement was quantified through the number of hours per week spent on the VSC platform, categorized into 1 to 3 hours, 4 to 6 hours, and 7 hours or more of participation. These data were extracted from VSC activity logs and reflect participation in structured online sessions (eg, classes and discussions) rather than passive internet use.

### Rationale for Self-Reported Measure

While objective cognitive measures (eg, neuropsychological assessments) were not included in this study, research has shown that subjective memory capability is strongly associated with cognitive performance and can serve as a reliable predictor of cognitive decline [35,36]. This study focused on perceived cognitive capability rather than objective performance, consistent with research emphasizing subjective well-being and self-perception in the digital health context [8].

### Statistical Analysis and Power Considerations

To enhance the robustness of the estimates, bias-corrected bootstrapping was used, a technique chosen for its ability to provide more accurate CIs in path analysis [37]. This procedure, based on 5000 bootstrap samples, has been shown to provide more accurate CI estimates in mediation and path models than percentile bootstrap methods [38,39]. Further, it allows for indirect effects within the model without type 1 errors, which are often associated with percentile bootstrap methods [37,40]. In the path analysis, standardized path coefficients and explained variance (*R*^2^) were reported to describe the strength and explanatory contribution of the modeled associations [41,42]. Effect size indexes designed for mean differences (eg, Cohen *d*) were not applicable because the analyses focused on associations among continuous variables rather than group comparisons.

## Results

### Overview

Table 1 shows that the participants’ average age was 77.86 (SD 11.55; range 47-98) years. The sample was approximately 72.1% (31/43) White individuals, 23.3% (10/43) African Americans, and 4.7% (2/43) Asian individuals. Only 1.9% (1/53) of the participants stated that they were Hispanic. Most participants (41/50, 82%) were female, and male individuals accounted for 18% (9/50). Participants’ educational levels were as follows: 34% (16/47) had a 2-year college degree, 25.5% (12/47) had a master’s degree, 25.5% (12/47) had a high school diploma, and 14.9% (7/47) had a bachelor’s degree. Most participants maintained their social network or contact via phone (34/53, 64.2%), and 28.3% (15/53) reported engaging with others mainly through the VSC program. Only 7.5% (4/53) of the participants reported engaging with others in person.

The average score on the MIA capacity subscale [25] was 39.00 (SD 10.06; range 14-61). The average score on the UCLA Loneliness Scale was 7.69 (SD 3.60; range 0-12), and the mean score on the Older People’s Quality of Life Questionnaire was 13.22 (SD 3.45; range 3-19).

**Table 1.** Sample demographics (N=53).

	Values
Age (y), mean (SD; range)	77.86 (11.55; 47‐98)
Gender (n=50), n (%)	
Male	9 (18)
Female	41 (82)
Race (n=43), n (%)	
Asian	2 (4.7)
Black or African American	10 (23.3)
White	31 (72.1)
Educational level (n=47), n (%)	
High school diploma	12 (25.5)
2-y college degree	16 (34)
Bachelor’s degree	7 (14.9)
Master’s degree	12 (25.5)
Socialization tool, n (%)	
Phone	34 (64.2)
VSC^a^	15 (28.3)
In person	4 (7.5)
OPQOL^b^ score (quality of life), mean (SD; range)	7.69 (3.60; 0-12)
UCLA^c^ Loneliness Scale score (reverse scored), mean (SD; range)	13.22 (3.45; 3-19)

a VSC: Virtual Senior Center.

b OPQOL: Older People’s Quality of Life Questionnaire.

c UCLA: University of California, Los Angeles.

### Path Analysis

**Figure 2.**
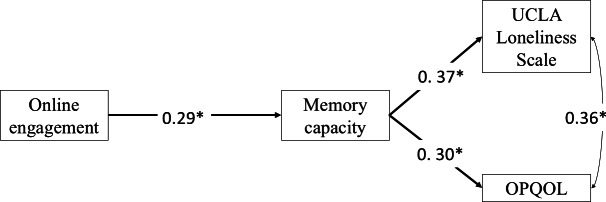
Path analysis with indirect effect. *P<.05. OPQOL: Older People’s Quality of Life Questionnaire; UCLA: University of California, Los Angeles.

Path analysis revealed, as Figure 2 shows, that online engagement significantly increased older adults’ subjective memory capability (β=0.29, 95% CI 0.08-0.50; *P*<.02). Individuals’ memory capability was positively associated with both quality of life (β=0.90, 95% CI 0.09‐0.57; *P*<.0001) and loneliness (β=0.37, 95% CI 0.18-0.57; *P*<.003). While demographic factors did not significantly influence participation in the VSC program, there were no direct effects on online engagement and quality of life and loneliness (refer to the full report in Table 2).

**Table 2.** Path analysis: effect of online engagement on loneliness and quality of life.

Variable	Coefficient (95% CI)	β (StdYX; 95% CI)
Age→online engagement	0.003 (−0.01 to 0.01)	0.05 (−0.19 to 0.29)
Gender→online engagement	1.19 (−0.09 to 2.48)	0.20 (−0.01 to 0.42)
Years of education→online engagement	0.02 (−0.08 to 0.13)	0.05 (−0.18 to 0.28)
Ethnicity→online engagement	1.77 (−2.26 to 5.18)	0.09 (−0.12 to 0.32)
Online engagement→memory capability	1.65^a^ (0.44 to 2.86)	0.29^a^ (0.08 to 0.50)
Memory capability→loneliness	0.07^a^ (0.03 to 0.12)	0.37^a^ (0.18 to 0.57)
Memory capability→quality of life	0.07^a^ (0.02 to 0.12)	0.30^a^ (0.09 to 0.50)

a *P*<.05.

## Discussion

### Interpreting the Role of Subjective Memory Capability

This study examined the association between structured online engagement and subjective memory capability among older adults participating in a VSC program. The findings suggest that increased participation in VSC activities was positively associated with subjective memory capability, which, in turn, was significantly linked to higher quality of life and reduced loneliness. However, online engagement was not directly associated with well-being measures, indicating that subjective memory capability plays a key mediating role.

A central finding of this study is the indirect effect of online engagement on well-being through subjective memory capability. While previous research has examined the benefits of online engagement for reducing social isolation and loneliness [8,11], our findings highlight a different pathway. Online engagement may be associated with cognitive self-perception, which may, in turn, relate to emotional and social well-being.

This aligns with prior studies showing that a positive self-view of memory capability can improve confidence in cognitive abilities, leading to greater social participation and engagement in intellectually stimulating activities [1,35]. The VSC program may provide an environment where older adults can reinforce their cognitive self-efficacy through structured interactions, online discussions, and virtual socialization.

Given the increasing emphasis on digital health interventions for aging populations, this study underscores the need for more tailored cognitive engagement programs that explicitly target memory self-perception and self-efficacy rather than focusing solely on social connectedness.

### Comparisons With Prior Digital Interventions

Our findings align with results from the I-CONECT study that demonstrated that internet-based social interaction interventions can enhance cognitive functions in older adults [13,14]. Similarly, research on cognitive training apps and digital learning platforms has suggested that active digital engagement can reinforce cognitive reserve and maintain subjective cognitive function in aging populations [3,6].

However, this study differs in its approach by focusing on a blended intervention integrating social and cognitive engagement. Unlike traditional cognitive training programs emphasizing memory exercises or problem-solving tasks, the VSC model incorporates structured discussions, interactive virtual activities, and peer-to-peer engagement. This approach may provide a more sustainable and engaging format for older adults, particularly those who may not actively seek cognitive training but can benefit from embedded cognitive engagement within social participation. These findings suggest that digital health interventions targeting cognitive well-being should combine socialization, structured engagement, and cognitive stimulation to maximize effectiveness [7,8].

### Implications for Digital Health and Aging Care

The results have several implications for digital health initiatives and gerontological practice. Structured online engagement programs such as the VSC could be incorporated into broader telemedicine and aging care strategies to support older adults in maintaining cognitive and social well-being. Given the growing prevalence of digital health platforms, integrating online engagement as a recognized intervention in aging services may help address social isolation and support cognitive well-being among older adults, particularly those who are homebound or facing mobility challenges [9,17].

Beyond integration into health care, the findings also emphasize the need for more user-centered digital platforms designed specifically for older adults. Digital interventions should prioritize features that enhance cognitive self-efficacy, such as interactive discussions, virtual memory exercises, and personalized engagement experiences. Ensuring accessibility and ease of use is equally critical as digital literacy and technological barriers remain challenges for many older adults. Addressing disparities in digital access through training programs, affordable technology, and support networks will be essential in expanding the reach and effectiveness of online engagement programs [18,19,43].

### Limitations and Future Research

While this study provides valuable insights, several limitations should be considered. The cross-sectional design limits the ability to establish causality among online engagement, subjective memory capability, and well-being. Future research using longitudinal data would clarify how sustained digital participation influences cognitive and emotional outcomes over time. Additionally, while subjective memory capability is a widely used measure in cognitive aging research, incorporating objective cognitive assessments would strengthen the findings and allow for a more comprehensive evaluation of the impact of online engagement [36,44].

The relatively small sample size presents another limitation as it restricts the generalizability of the findings to broader populations of older adults. Given the modest sample size, the findings should be interpreted as exploratory and hypothesis generating, and replication in larger samples is warranted. Future research should also explore comparative analyses of different online engagement models, including cognitive training applications, telehealth platforms, and interactive digital communities, to determine which approaches are most effective in supporting cognitive and social well-being.

### Conclusions

This study examined the potential of structured online engagement programs to enhance cognitive self-perception, influencing emotional and social well-being among older adults. The findings reinforce the importance of subjective memory capability as a key mediator in the relationship between digital engagement and overall well-being. As digital health and telemedicine evolve, integrating interactive and cognitively engaging online experiences into aging care models may provide scalable solutions for promoting cognitive and social health in older populations. Expanding access to well-designed and inclusive digital programs will ensure that older adults can fully benefit from the advancements in digital health and online interventions.

## Abbreviations

I-CONECT: Internet-Based Conversational Engagement
MIA: Metamemory in Adulthood
UCLA: University of California, Los Angeles
VSC: Virtual Senior Center
